# Green Solvents for Lipid Extraction From Microalgae to Produce Biodiesel

**DOI:** 10.3389/fchem.2022.884274

**Published:** 2022-05-18

**Authors:** Xiaofang Liu, Dayong Yu, Hangyu Luo, Can Li

**Affiliations:** Guizhou Provincial Key Laboratory for Rare Animal and Economic Insects of the Mountainous Region, College of Biology and Environmental Engineering, Guiyang University, Guiyang, China

**Keywords:** microalgae, biodiesel, lipid extraction, deep eutectic solvents, green solvent

## Abstract

Microalgae are considered as the third-generation feedstock for biodiesel production, and lipid extraction plays a significant role in efficient production of biofuels. Numerous technologies including chemical, mechanical, and biological have been achieved but high efficiency and potential application on an industrial scale are still needed. This review discusses the factors that influence biodiesel quality and the relative green and sustainable solvents for lipid extraction.

## Introduction

With the concern that fossil fuels have caused global warming and an energy crisis, there is a need to diminish the dependence on it and explore renewable energy. Biodiesel is considered as a potential alternative to petro-diesel as it is non-toxic, biodegradable, has an enhanced cetane number, higher flash point, is renewable, and is produced by transesterification of renewable feedstocks, resulting in monoalkyl esters from fatty acids (Meher et al., 2004; Hoekman et al., 2012; Fazal et al., 2013; Pan et al., 2022) In the past 2 decades, three generations of biodiesels have been investigated and each generation had advantages and disadvantages over different feedstocks. Table 1 provided the summary of the generations of biodiesels. High-efficiency converted algae for biodiesel urgently needs to be explored.

**TABLE 1:** Differences of three generations of biodiesel.

Biodiesel	Feedstock	Advantage	Disadvantage	Processing Technology	Ref
First Generation	Edible Plant Seeds	Relative high yield	negative impact on the arable land, food and environment	Esterification and Transesterification of oils	Ahmad et al. (2011)
					Mancaruso et al. (2011)
Second Generation	Non-edible Plant Seeds, Waste Cooking Oil, Lignocellulosic Feedstocks	environmentally friendly, higher cetane number, clean and renewable properties	inabundant reserves, poor property in cold temperatures, greater amount of saturated fatty acids	Esterification and transesterification of oils/seeds (utilises organic catalyst/additives)	Ahmad et al. (2011)
	Animal Fats				Bhuiya et al. (2014)
Third Generation	Algae (especially Microalgae)	high growth rate and lipid contents, lower demand for water and land, High efficiency in fixing CO_2_	dependence on light, complex and inexpensive procedures to produce biodiesel	Cultivation, harvesting, lipid extraction, transesterification	Ahmad et al. (2011)
					Alaswad et al. (2015)
					Saladini et al. (2016)

## The Production of Biodiesel From Microalgae

### Fuel Properties Parameters of Biodiesels

Generally, factors including the viscosity, oxidation stability, cetane number (CN), cold filter plugging point, flash point, saponification value (SV), energy density, and density of biodiesel are determined by the fatty acid composition, which plays a crucially important role in biodiesel qualities. How to optimize the parameters with technologies to enhance quality during the production process is a key question.

Fatty acids are comprised of unsaturated, namely mono-unsaturated (denoted as Cn:1) and polyunsaturated (Cn:2 or 3), and saturated (Cn:0) fatty acids. Viscosity increases along with the chain length and fatty acid saturability. Transesterification, also called alcoholysis, of the algae oil to the corresponding fatty ester (biodiesel) is the most promising approach to the high viscosity problem (Demirbas, 2009). Better oxidation stability, meanwhile, requires a high level of fatty acid saturation (Graboski and McCormick, 1998). CN increases with the enhancement in chain length and fatty acid saturation level (Içingür and Altiparmak, 2003; Knothe, 2005). The higher the saturation degree is, the poorer the cold filter plugging point is (Ramos et al., 2009). A shorter chain length provides a lower flash point and the density will be high when the polyunsaturation level is high (Karmakar et al., 2010). Fatty acid methyl esters with a carbon chain length from 12 to 20 are identified as biodiesel. The SV indicates the chain length of triglycerides and explains the content of free fatty acids, high levels of which can be reduced by acid catalysts (Srivastava and Prasad, 2000; Aransiola et al., 2010).

### The Influence of Reaction Factors on Biodiesel Derived From Microalgae

The effect of water content mainly refers to the handled dry and original wet algal biomass (Atadashi et al., 2012) to produce biodiesel. The presence of water plays a crucial part in triglyceride hydrolyzing to free fatty acid (FFA) resulting in soap and emulsions formation, hence the water content control is lower than 0.05% (w/w) (Sanford et al., 2009). Another dimension, a high water content of up to about 98%, generates the hydrated shell around algal cells affecting energy as well as mass transfer (Martinez Guerra et al., 2018), furthermore, posing difficulty in the extraction of lipids.

Although homogeneous acid and base catalysts exhibit high efficiency and universality, the separation is tough and requires further neutralization. Although homogenous base-catalyzed reaction is 400 times quicker than the acid-catalyzed reaction, acidic catalysts are normally used for the feedstocks with high contents of FFAs and water (Aransiola et al., 2010), while the alkaline ones are very sensitive to them, affecting the introduction to the laboratory and the industrial popularly (Frascari et al., 2008). A heterogeneous catalyst is easily separated, reducing the cost of catalyst recovery (Tran et al., 2017; Zhang et al., 2019), and the inexpensive basic catalyst including calcium oxide, calcium hydroxide, and magnesium oxide also reduce the environmental impact (Zhang et al., 2010).

Biodiesel can be synthesized from algae through a traditional two-step method (oil extracted from the algae and then transesterified into biodiesel) or an *in-situ* approach (extraction of oil, esterification of FFAs, and transesterification of triglycerides occur simultaneously) (Sara et al., 2016; Martinez-Guerra et al., 2018; Al-Ameri and Al-Zuhair, 2019). The former requires a long time, a large reactor, and an even higher investment, while the latter offers an efficient method, which simplifies the production process, minimizes the dosage of solvents, and can give improved biodiesel yield.

## The Ionic Liquids for Enhancing Lipid Recovery for Biodiesel Preparation

Ionic liquids (ILs) are widely known as green organic solvents and are non-aqueous salt composed of organic cations and organic or inorganic anions melting at low temperatures (<100°C). ILs are suitable for lipid extraction owing to the major advantages:

1)eco-friendly in nature (Zhao et al., 2019); 2)non-volatile and non-flammable (Vekariya, 2017; Harris et al., 2018); 3) good thermal and chemical stability (Khiratkar et al., 2018); 4)synthetic flexibility (Kim et al., 2012); and 5)immiscibility with organic solvents (Li et al., 2013).

**FIGURE 1:**
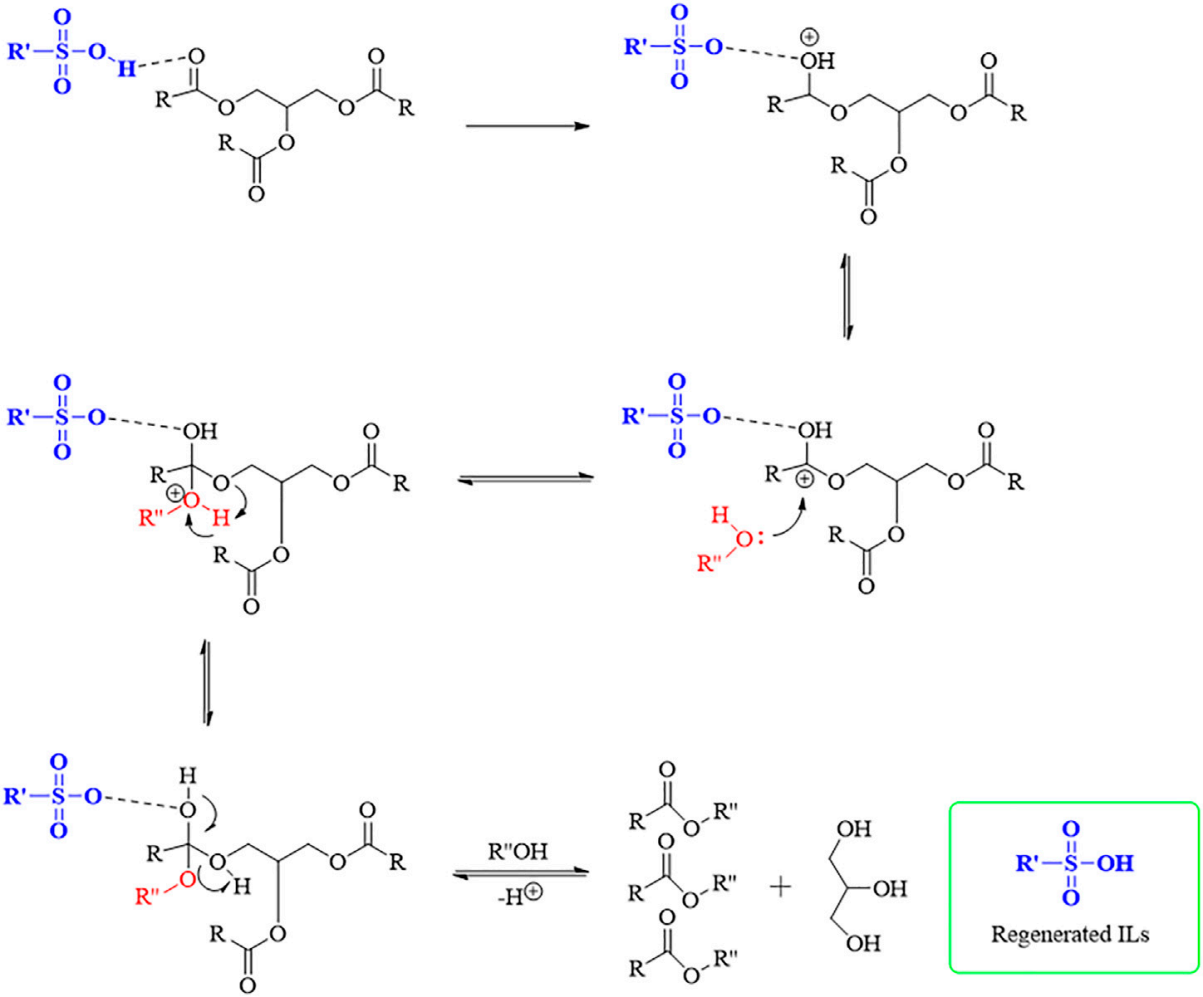
Plausible mechanism of transesterification of microalgal lipids with alcohol using sulfonic ILs catalyst [Here, R denotes the alkyl chain of the triglycerides, R′ denotes the struture of the ILs expert the -SO3H group and R’’denotes the alkyl group of alcohol].

The plausible mechanism of the transesterification of microalgal lipids with alcohol using sulfonic ILs catalyst is shown in Figure 1.

### Conventional Ionic Liquids

The research explored the lipid extraction effect of [Bmim][MeSO_4_] from *Chlorella vulgaris* combined with ultrasound pre-treatment and drew a comparison to the traditional Soxhlet method and Bligh and Dyer’s method. Results demonstrated that IL exhibited 2-fold and 1.6-fold higher lipid extraction than the classic approaches (Kim et al., 2013). Similarly, Choi compared the lipid extraction yield of *Chlorella vulgaris* by the mixture of ILs, with the assistance of organic solvents. They confirmed that lipid extraction yield was enhanced using IL mixtures, which was ascribed to the synergistic effects with different anions (Choi et al., 2014). The introduction of ILs on wet algal biomass has proven to be the easiest and most efficient method of lipid extraction (Orr and Rehmann, 2016). Furthermore, the poly-ILs catalyst with a large surface area and abundant mesopores have also been investigated for biodiesel preparation (Bian et al., 2020). The combination of magnetic nanoparticles (MNPs) and ILs was used to separate microalgae from the aqueous phase with 99% efficiency and 99% lipids extraction efficiency under ILs/hexane, respectively (Egesa and Plucinski 2022).

Due to ILs’ unrealistic application at an industrial scale due to costs and environmental impact, limited articles are available in the literature on the synthesis of biodiesel (Motlagh et al., 2019). ILs have been confirmed to not be harmful for humans, but the preparation routes involve processes that require expensive, toxic, and volatile reagents (Harris et al., 2018; Singh and Savoy, 2020).

### Deep Eutectic Solvents

DESs are generally comprised of organic salts (such as choline chloride, choline acetate, quaternary ammonium salt, or phosphonium salt) and hydrogen-bond donors (HBD) (such as amides, amines, alcohols, and carboxylic acids) that are stable in hydrogen bond interactions, with a melting point lower than that of anionic and cationic counterparts (Zhang et al., 2010; Durand et al., 2013). As a novel class of renewable solvents, DESs emerge with several benefits including low-cost synthesis, non-toxicity, low volatility, and high biodegradability (Zhang et al., 2010; Radošević et al., 2015).

The investigation reported that the cell wall of *Chlorella* sp. and *Chlorococcum* sp. contains α-cellulose, hemicellulose, protein, lipid, and ash (Loos and Meindl, 1982). tThe combination of DESs and α-cellulose, hemicellulose, affords new hydrogen bonds that could damage the microalgae cells to enhance the lipid extraction. Three different DESs, aqueous choline chloride-oxalic acid (aCh-O), aqueous choline chloride-ethylene glycol (aCh-EG), and aqueous urea-acetamide (aU-A), were applied to pretreated *Chlorella* sp. and the lipid recovery rate of biomass was evaluated. Results demonstrated that the lipid recovery rate was enhanced from 52.0% of a blank control group to 80.9, 66.9, and 75.3% of the biomass treated by aCh-O, aCh-EG, and aU-A, respectively (Lu et al., 2016). There a consistent conclusion obtained when DESs are treated on wet and unbroken (water content is 65–67%) with *Chlorella* sp. and *Chlorococcum* sp. (GN38) through one-step and two-step methods (Pan et al., 2017).

## Conclusion and Perspectives

The review discusses the factors that influence biodiesel quality and conversion of microalgal. It is necessary to adjust these technical parameters with analysis to ensure the feasibility of biodiesel production. The main aims of green solvents for extraction should be eco-friendliness, less dosage of solvent, increasing the quality of the product without byproducts, and saving energy. The efficient DESs with suitable organic salts and HBD to extract lipid are in demand. Microalgae research and development are expansive and synthesis technology for biodiesel from microalgae still requires much investigation. The life cycle analysis of the existing processes will be beneficial for commercial application.
